# National Women’s Health Plan, Selected Countries Experiences and Necessity of Developing It in Iran: A Narrative Review Article

**Published:** 2019-01

**Authors:** Batoul AHMADI, Sedigheh SALAVATI

**Affiliations:** 1. Department of Health Management and Economics, School of Public Health, Tehran University of Medical Sciences, Tehran, Iran; 2. Department of Public Health, School of Nursery, Maragheh University of Medical Sciences, Maragheh, Iran

**Keywords:** Women health, National health plan, Iran

## Abstract

**Background:**

National plans have an important role in defining vision, goals, priorities, and action plans. The present paper examined the international experience in developing upstream documents concerning women’s health; it regards the necessity of developing Iranian women’s health plan.

**Methods:**

This review, conducted with search in electronic databases and literature of no limitation in terms of years conducted. The phrases such as “woman health policy”, “woman health promotion”, “woman health strategy” and “woman health plan” were searched. The criteria based on which the countries were chosen were the degree to which they were developed in health system, health indexes, the accessibility of required information, and the possibility of benchmarking the new methods.

**Results:**

Emphasize on gender as a determinant of health, increasing the economic activities of women, decreasing the violence against women, gender-based researches, and inter-sectorial approach are some common items in the women’s health plans in selected countries. The main upstream documents about women health in Iran such as ‘Research policies and principles of women issues’, “Women Charter of Rights in Iran” were not developed and implemented in form of a comprehensive plan so we need to formulate a full women health national plan that summarizes all previous policies with addition of new needed strategies inclusion.

**Conclusion:**

Developing a national plan for Iranian women’s health regarding with selected countries experiences makes a long-term vision for health and obtains the institutional and organizational efforts and resources necessary for women health promotion in Iran.

## Introduction

Policies, strategies, and national programs have important role in defining the priorities, decisions, and actions to improve and maintain the population health (1).

The aim of developing strategies, policies and programs is to determine the priorities of the country based on the basic needs of health and the better use of the existing resources (2). In most of the countries which have “national health plan”, the concepts of perspective, policy, strategy, and program are included in this national plan (3).

The national health policy is comprised of three main components. Firstly, there are structural factors which include public interventions to create, maintain, and strengthen cultural, economic, social, and political determinants. The second component is the lifestyle determinations which include interventions whose aims are people and they focus on the changes in individuals’ behavior and lifestyle. These determinants are more manageable, and in contrast to structural determinants, they are easier to handle. The third component of a national health plan is the social and empowering determinants which provide the link between lifestyle and structural determinants (4). Women’s health is the infrastructure of family and society’s health, and it is one of the most important concepts in socio-economic development and welfare (5, 6). Today, women’s physical, mental, social health model is emphasized identified by three key points (7, 8):

1. Health is not necessarily the lack of disease, and it included physical, mental, and social health
2. Women’s health is multidimensional, and reproductive health is only part of it.
3. Women’s health is influenced by social, cultural, mental, and biological factors.

In Iran, despite the improvements in the field of health since the victory of Islamic Revolution, we face challenges about women’s health. For instance, mortality profile study in 29 provinces in 2007 showed that breast cancer is increasing fast, and it is the sixth cause of mortality in women in Iran. Today, the incidence of breast cancer in women occurs ten years earlier than other countries (9). Only 19.62% of women from 15 to 24 have suitable awareness about how to prevent HIV (10). About 59% of women are overweight, and more than 20% of middle-aged women have dyslipidemia. More than 25% of elderly or middle-aged women have blood pressure, and more than 20% have diabetes. Tobacco use has also a high growth in women, and girls’ average age of smoking has decreased. There are challenges in research related to women’s health such as lack of interdisciplinary researches, lack of coherent information system and access to genuine statistics, the weakness of analysis approach, and lack of enough connections among researchers of women’s health, insufficient research budget, and lack of need assessments at different levels (11). In comparison to developed countries, the gender gap is significant in Iran (12). In the field of gender equality among other selected Middle-East countries in 2011, Iran had the 7^th^ rank in the area of women economic participation, the 9^th^ rank in educational achievements, the 6^th^ rank in the health and survival, the 10^th^ rank in political empowerment, and the 11^th^ rank in the average rating on these four indicators (13).

With regard to the mentioned points, the significance of planning and policy-making related women’s health becomes clear. In Iran, some upstream documents such as “Research policies and principles of women issues”, “Women Charter of Rights and Responsibilities” and “Main Women Health Strategies” were developed but each of them addresses some aspects of women’s health and no one has a comprehensive approach to women’s health planning. Moreover, no scientific and systemic method were used for developing these plans.

Comprehensive and strategic plan for women’s health can create a long-term vision for improving health in the community, and it will answer the questions of where to go based on weaknesses, strength, threats, and opportunities, what should be done, and how we are going to reach the place we are supposed to. Action plans will create political commitment so that political interest and necessary resources are created to implement the long-term health plan (7). Studying the experiences of other countries in the area of women’s health policymaking can suggest recommendations for drafting a coherent national plan for women health in Iran.

The present paper examined the international experience in developing upstream documents concerning women’s health, and by looking at these documents, it regards the necessity of developing a national women’s health program.

## Methods

This narrative review was conducted by searching the Internet resources. Scientific resources, articles, scientific reports, national and international documents in the field of women’s health, programs, strategies, and policies were reviewed without any time limitation. Other women’s health documents were extracted from women’s health-related websites of institutions and organizations in these countries. Searching the English databases, including PubMed, Science Direct, and Google Scholar, and Persian databases, including Iran Medex, SID was done. Moreover, the search for English and Persian phrases such as “woman health policy”, “woman health promotion”, “woman health strategy” and “woman health plan” was used. To extract upstream documents, websites of the relevant institutions and bodies, including the Ministry of Health and Medical Education, Islamic Parliament of Iran, Department of Women and Family Affairs and etc. were searched.

The criterion, based on which the articles and documents were chosen, was the most possible in accordance with the aim of this research. The criteria based on which the countries were chosen were the degree to which they were developed in health system, health indexes, the accessibility of required information, and the possibility of benchmarking and implications the new methods.

## Results

The experiences of European countries, United States, Australia, Canada, and New Zealand in the institutional actions and developing the upstream documents on women’s health were studied in this article.

### European countries

The non-governmental institution of women’s health was founded in 1996 to enhance gender equity in health, to conduct studies, and to make social policies. This organization put gender mainstreaming in its agenda, and it has annual plans. For example, some priorities of its women health plan for 2018–2020 include (14):

- Interventions for the prevention of chronic diseases
- Healthy and active aging for throughout life
- Paying attention to sex and gender in medical education
- Implementation of sex and gender in all relevant social policies

In addition to this institution’s efforts, WHO Europe region has also drafted a strategic plan of women’s health in 2001 (15). This plan emphasizes the concentration of the membered countries on reviewing the previous and present policies regarding women’s health issues. In this program, identification of social and economic backgrounds to form the foundation of women’s health is noted. One of the key elements of women’s health action plan throughout the European countries is to use lifetime approach in strategies of women’s health, and in fact, to pay attention to preserve women’s health before birth to the old ages.

The other important element is to increase the participation of women in their own health promotion. The improvement of the position of women as the actors and decision-makers in the area of women health will lead to the consideration of their voices and preferences in policy-making and prioritization. Using comprehensive policies to prevent violence against women is the other important thing in the action plans. Other elements which are to be placed in the action plans are doing researches based on sex and gender, statistics and data gathering based on sex, and reviewing the methodology and financial support of clinical trial studies to ensure the inclusion of women in these studies (15).

### The United States

“A vision for 2020 for women’s health research” is the title of the document published by research institution of US women’s health. The aim of this document is increasing the researches with regard to the sexual differences in basic sciences, regarding the differences between gender and sex in designing and implementing the medical technologies, designing prevention and diagnosis services specific to women and girls, promotion of strategic efforts for making use of the result of women’s health researches, the function of social networks for the promotion of women’s health, and finally the improvement of human resources education necessary to conduct the women health researches (16).

Besides the activities of this institution, Women’s Health Department, the subset of ministry of Health and Human Services in United States, developed the strategic plan of 2010–2015 which covers the following aims (17):

- Developing leadership and coordination policies for local, state, and federal levels to make a national health policy for women and to increase the cooperation between health organizations, other institutions, and people.
- Implementation, evaluation, and correction of the women and mothers’ health programs in the area of the problems ignored by private and state institutions at the national level.
- The provision of evidence about inequalities in prevention, diagnosis, and treatment of diseases among women.

In line with developing programs and upstream documents of the US, each state has tried to draft women’s health plan. Maryland strategic plan for women’s health in 2001–2006 is one of them. One of the noticeable strategies in this plan was to link national and state organizations and governmental and non-governmental institutions to decrease the inequalities in access the health services for women. According to local needs, developing community-based services to provide comprehensive and integrated services for women is emphasized. Trying to develop the women’s health topics in medical education curriculums is another important strategy.

Women and children’s health in Arizona, another state of the US, drafted another strategic plan for women and children’s health for 2011–2015. This program focused on decrease in women and children’s mortality, decrease in health Outputs inequalities, and increases the health and accessibility to health services. This document was drafted after the need assessment of women and children’s health and the promotion of men’s participation in the improvement of women’s health, reduce the damages of violence through implementing approaches to identify the victims and designing preventive programs are emphasized (18).

### Australia

In 1989, the first national women’s health policy was drafted in Australia with the title of “Women’s Health in a Changing Society”. This national policy includes 5 different operational areas: improvement of the women’s health services, gathering data and doing researches in the area of women’s health, improvement of women’s participation in the health decision makings, and training of the health workforce to provide the gender-sensitive services.

This national policy got the basis for planning the women’s health services in all the levels of the government two decades later. One of the achievements of this policy was developing the women’s health centers, which led to developing a longitudinal study of women’ health in Australia, examined the health of more than 400000 women for a period of 20 years. This set of studies study on different aspects of women’s lives such as physical, mental, emotional aspects, reproduction, malnutrition and obesity, income, and economic condition up until old age.

National policy of Australian women’s health in 2010 was developed by Department of Health and Ageing to follow and complete the previous national policy by regarding the results of conducted surveys. Goals were developed in this national policy especially considered these points:

- Gender as a determinant of women’s health
- Difference health needs of women at different stages of their lives
- Giving priority to the needs of the women who are most at risk of a poorer health
- Ensuring responsiveness of health system to all women with a focus on prevention and health promotion
- Supporting effective and coordinated research and knowledge translation to strength the evidence-based decision-making

Different regions in Australia have tried to design the action plans considering the national policies (19–21).

### Canada

The first movements about improvement of women’s health were formed in Canada in 1970s and 1980s and some women from across the country were gathered to each other in the form of discussion groups or educational forums to share their experiences and knowledge. These movements synchronized with efforts of other groups such as consumers’ rights protection groups and women’s rights and finally the Canadian Women’s Health Network was formed at 1993 (22). This network is a national volunteer organization to improve women’s health which provides reliable information and researches about women’s health and it is trying to change unfair health policies and plays role of knowledge transfer (23).

Along with social movements and institutions and following the encouragement of countries by United Nations to develop strategies of reducing gender inequalities, the federal government of Canada developed women’s health strategy in 1999 with these purposes (24):

- Ensuring responsiveness of all policies and programs against gender differences and meet women’s health needs
- Increase the knowledge about women’s health needs
- Providing effective health care services for women
- Improvement of women’s health through prevention measures and reduction of risk factors of women’s health

In Canada, a non-profit foundation called Canadian Women’s Health also is active in promoting women’s sexual and reproductive health. This foundation supplies some researches about improvement the financing of Canadian women’s health. In addition, it participates in financing of educational programs for healthcare professional in order to improve mothers and children health and reduce the incidence of cervical cancer (25). Some of strategies developed by this foundation with focus on improvement of Canadian immigrant women’s health in 2006 were (26):

■ Adopting an inter-sectoral approach for long-term and effective improvement of women’s health
■ Strengthening institutions and organizations which are active in the field of women’s health in order to more reflection of needs and women’s expectations in policies
■ Attention to social determinants of health
■ Gender-based studies for women’s health policy-making

One of gender-based reports in Canada was published by Canada’s Bureau of Statistics of Ministry of Industry in 2016 which provided a picture of women’s health based on indexes of social determinants of health, behavioral and mental health, nutrition health, physical activity, food security, prevalence of chronic diseases, and injuries and hospitalizations (27).

### New Zealand

New Zealand’s Ministry of Women was formed as a governmental department in 1984. Origin of creating Ministry of Women is from women’s freedom movement in 1970 which was with the aim of increasing women’s politic power. The main purpose of the Ministry is equality of access to power, resources, and opportunities for women and is active in the four main fields including training women, improvement of utilization of women’s skills, deal with violence and increase of women’s participation and leadership (28). Ministry of Women provides some annual reports to evaluate improvement of women’s status and monitor these four main fields and uses its results to develop programs (28).

Some non-governmental organizations are also active about improvement of quality of New Zealand women’s lives that the most important of them is institution of women’s health movement. Role of this institution is providing consultation to professionals and health policymakers and other non-profit organizations in line with improving women’s human rights in the health area and providing information and educational services for women to enable them (29).

In New Zealand, the importance of attention to gender in policy-makings became clear after publishing gender inequalities reports in health by Ministry of Health (30). Following that the New Zealand women’s health organization developed national women’s health strategy based on sex and gender as determinants of health, considered the following principles in the strategies:

- Developing health strategies based on sexual and reproductive health, medical confidentiality, access to education and information, poverty reduction, and health priorities of vulnerable groups
- Using gender analysis approach in researches and studies
- Considering the age, racial, physical ability, income and geographical location diversities in women’s health policy making
- Emphasis on inter-sectoral approach in prevention and health promotion because all sectors should pay attention to effects of decisions and policies on women’s health.
- Having the Holistic approach based on whole periods of women’s lives in developing the policies and attention to women’s roles and experiences from birth to death (31).

Table 1 shows a short of institutional and programming measures and the emphasized points of each institution and program in the countries.

**Table 1:** Actions and plans related to women health in selected countries

***Country***	***Main actions and plans in field of women health***	***Areas of focus of actions and plans***
Australia	- Developing the national health policy for women as a foundation for future policies and programs - Conducting series of surveys about women health - Evaluation of national health policy for women for a 20 years’ time period. - Developing national health policies based on results of previous policies evaluations and surveys.	- Prevention from acute diseases via control of risk factors - Promotion the sexual, productive and mental health - Promotion the elderly women health - Attention to gender as an important determinant of health - Emphasize on women health promotion throughout their whole life - Assurance the responsiveness of system for all groups of women - Support from the comprehensive and coordinated research and knowledge transition for improvement the evidence about women health
New Zealand	- Formation of the ministry of women - Publishing the universally reports about evaluation of women health by ministry of women - Formation the non-governmental consultation institution in the women health area - Activities of non-governmental institutions for provision the consultation services about women health policy making	- Educational services to women for improvement their skills in economic contributions - Trying to decrease the violence against women - Increasing the chance of women leadership in different fields in the community - trying to increase the gender equity - improving equity between different groups of women - focus on prevention and health promotion with emphasize on inter-sectoral approach - using the evidence in developing the women health strategies - considering the throughout lifespan in women health policy makings - strategic approach to developing the women health plan
European countries	- establishment the non-governmental institution of women’s health - developing the universal women health plans - the strategic planning for women health by regional office of world health organization for Europe - establishment the national coordination commit-tees of women health in the member states	-prevention from acute diseases - trying to develop gender-sensitive health systems - attention to throughout the lifespan approach in developing the plans -increasing the women participation in health promotion -promotion the women’s position in decision-making settings -organization the women’s rights groups -providing the services based on health needs and evaluate the outcomes - considering the confidentiality in providing health care services for women -implementing the comprehensive policies for protection of women against violence - conducting researches considering with sex and gender -men participation in improving the women health - encouraging the self-care - identifying the social and economic determinants of health
US	- developing the upstream document for women’s health research - developing the strategic plan by department of women health - creating the women health plans in the states level	-contribution the local, state and federal levels in developing the national health policy for women - Developing a comprehensive framework for evaluation the women health and life - Gathering and analysis data based on gender and sex - Gathering and analysis data about social determinants of women health in the states level - Increasing the men participation in women health improvement - Decreasing damages of violence
Canada	- Developing the women health networks - Establishment of the nonprofit foundation of Canadian women health - Developing strategies for immigrant women health in Canada - Publishing the gender-sensitive report of Canadian women health	- researches about women’s health for informing the policymakers from evidence - Implementing inter-sectorial approach in women health promotion - reflect the women’s needs and expectations in policy makings via Expansion the institutions and organizations which are active in the field of women health - Analysis based on gender differences - Support from developing the networks, advocacies, and projects about women health promotion - Improvement in the community-based researches aim to women health promotion in various dimensions

### The upstream documents about women’s health in Islamic Republic of Iran

- “Research policies and principles of women issues” were developed by Supreme Cultural Revolution Council in 2000 (32).
- “Women Charter of Rights and Responsibilities in Islamic Republic of Iran” approved as a reference document in policy making in cultural and social affairs in 2004 in suggestion of Social and Cultural Council of Women (33).
- “Women’s health promotion policies and strategies” approved by Supreme Cultural Revolution Council in 2007 (34).
- “Main Women Health Strategies in Islamic Republic of Iran” was published by office of women’s affairs in Ministry of Health in 2009.
- “Comprehensive plan of women and family development affairs” were approved by the government cabinet and then were sent to governmental executive organizations (35).
- “Health system transition roadmap” published by Iran Ministry of Health and Medical Education in 2011 (36).

## Discussion

The purpose of this research was identification of experiences of other countries about upstream documents and plans of women’s health and suggesting recommendations for drafting a national plan for women health in Iran after reviewing available documents related to women’s health in Iran.

Familiarity with experiences of other countries in codification of upstream documents about women’s health after attention to cultural, social and economic differences of countries can pave the way of codifying the national program of Iranian women’s health. For example, the strategic program of European women’s health emphasizes on importance of attention to women’s health in whole life duration. Moreover, collection of statistics and data based on sex and gender differences and designing health interventions in a way that to be sensitive on gender and needs of women’s health can be a notable point in patterning this document to develop women’s health program in Iran. In Australia, the national committees of coordination of women’s health are responsible to codify, execute and monitor operational programs (15) that this case mentioned in different documents of Iranian women’s health has been not considered a lot. Therefore one of important factors in codification of Iranian women’s health program can be adopting some policies to create structure of managing development of women’s health (37).

One of positive points about policy making of women’s health in Australia is launching survey based on women’s population during the past decades that provides evidence to policy-making (22). In Iran, conducting researches and providing scientific papers with centrality of women’s health and hygiene have been neglected a lot so that about forty percent of issues related to priorities of women’ health have been neglected relatively and also about forty percent have been neglected completely by them (38). Quantitative and qualitative promotion of evidence related to promotion of women’s health in developing national plan of women’s health should be considered.

Attention to women’s health in adopting policies of all sectors except the health sector is one of notable cases in national strategy of women’s health in New Zealand (31). Besides, we see participation of different groups of the society’s women in frame of public associations and organizations and in order to reflect women’s real needs in policies in reviewed countries (19, 21). Developing the national program of women’s health in Iran should integrate efforts of different organizations and institutions to promote women’s health and provide a framework for measure. The national program of women’ health is founded based on this belief that involvement and interference of all organizations and the society are necessary to promote comprehensive health of women and no organization or institution such as Ministry of Health will be able to solve challenges related to this issue lonely (39). The national health program should provide a framework to guide activities of all related institutions to promotion of women’ health, determine some purposes and strategies to response problems of women’s health in the specified period and meanwhile to be director of action plans in national and local levels.

In all selected countries attention to social determinants of women’s health, gender-based studies are notable cases in policy making of women’s health. Based on a quantitative study conducted in Iran a theoretical model and framework was provided for determinant factors of Iranian women’s health with considering national and cultural aspects that the most important aspects of that included biological, physical and social ability, lifestyle, familial support, familial responsibilities and relationships, social capital, work and life conditions, policies and structures and religion and spirituality with emphasis on Islam (40). Designing the national plan of women’s health in Iran is necessary for a way that all these elements and interactions among these components to be considered.

Evaluating the national documents with centrality of women’s health in Iran indicates attention of policymakers to importance of women’s health in our country during the past decades. One of these documents is about planning for research on women’s issues and defining the research principles in this regard in Iran (32). Other developed document defines the women responsibilities and rights in both individual and social levels (33).

Four other main upstream documents in Iran focus more on the women’s health and provide some policies and strategies to their health promotion (34–36) but despite presence of these documents in Iran and acceptable health promotion of women after victory of Islamic revolution, still, we are far from the ideal status. We did not have any comprehensive plan in Iran that considers different aspects and dimensions of women health. The developed upstream documents do not include the action plans and strategies via them the policies could be implemented. The current plans often do not have any executive and legal grantees for enforcement.

In developing our national plan we should include evaluation measures which systematically measure moving toward success to reformation of previous policies. The prerequisite of this is prediction of suitable indexes to evaluate and monitor the women’s health national plan in national, provincial and regional levels.

Developing the provincial and regional programs in line with the national plan of women’s health will have a crucial role in achieving goals of the national plan. Finally, evaluation of experiences of other countries shows importance of the following cases in developing women’s health plan in these countries that also can be noted by policymakers in the matter of women’s health national plan in Iran:

- Implementation of surveys based on women’s population in national and provincial level to produce evidence in order to policy making for women health in the country.
- Participation of the women in policy makings as the main beneficiaries through more participation in high level of decision-makings
- Pay attention to developing the action plans in lower levels of policy-making in line with the national program
- Pay attention to comprehensive physical, mental, social, economic and etc. health of women in their lifetime
- Making the policies and strategies in order to encourage men’s participation in promotion of women’s health
- Creating coordination women’s health committees in national and provincial levels in order to monitor and evaluate of women’s health in the provinces and determining needs of women’s health in each province
- Studies and analyses based on gender in planning related to providing services, researches, and surveys.
- Emphasis on gender equity in developing the programs and policies
- Pay attention to role of other sectors in addition to health sector in promotion of physical, mental, social, economic and etc. dimensions of women health

## Conclusion

Developing the national program of women’s health is necessary in a way that includes contents of the developed documents in previous years about health of women in the country and to collect efforts of all institution in a collection such as Supreme Cultural Revolution Council, Social and Cultural Council of Women, the Office of Women’s Affairs and in the Ministry of Health and the Department of Women and Family Affairs of the Presidential, Academy of Medical Sciences and experts in the field of women and women’s health. In this field using experiences of other leading countries will be helpful in developing of national women’s health program.

## Ethical considerations

Ethical issues (Including plagiarism, informed consent, misconduct, data fabrication and/or falsification, double publication and/or submission, redundancy, etc.) have been completely observed by the authors.
